# Effect of Photobiomodulation in Rescuing Lipopolysaccharide-Induced Dopaminergic Cell Loss in the Male Sprague–Dawley Rat

**DOI:** 10.3390/biom9080381

**Published:** 2019-08-19

**Authors:** Jayden A. O’Brien, Paul J. Austin

**Affiliations:** Discipline of Anatomy & Histology, School of Medical Sciences, Faculty of Medicine and Health, The University of Sydney, NSW 2006, Australia

**Keywords:** Lipopolysaccharide, low-level light therapy, microglia, neuroinflammation, Parkinson’s disease

## Abstract

Photobiomodulation (PBM) provides neuroprotection against dopaminergic cell death and associated motor deficits in rodent and primate models of Parkinson’s disease (PD). However, it has not yet been tested in the lipopolysaccharide (LPS) model of PD, which leads to dopaminergic cell death through microglia-evoked neuroinflammation. We investigated whether transcranial PBM could protect against dopaminergic cell death within the substantia nigra in male Sprague–Dawley rats following supranigral LPS injection. PBM fully protected rats from 10 µg LPS which would have otherwise caused 15% cell loss, but there was no significant neuroprotection at a 20 µg dose that led to a 50% lesion. Cell loss at this dose varied according to the precise site of injection and correlated with increased local numbers of highly inflammatory amoeboid microglia. Twenty microgram LPS caused motor deficits in the cylinder, adjusted stepping and rotarod tests that correlated with dopaminergic cell loss. While PBM caused no significant improvement at the group level, motor performance on all three tests no longer correlated with the lesion size caused by 20 µg LPS in PBM-treated rats, suggesting extranigral motor improvements in some animals. These results provide support for PBM as a successful neuroprotective therapy against the inflammatory component of early PD, provided inflammation has not reached a devastating level, as well as potential benefits in other motor circuitries.

## 1. Introduction

Parkinson’s disease (PD) is the second most common neurodegenerative disorder, characterised by motor deficits such as resting tremor, rigidity, and difficulty in initiating and sustaining voluntary movement [1]. These symptoms worsen as the disease advances primarily due to the progressive death of dopaminergic neurons in the substantia nigra pars compacta (SNc). A large degree of cell loss in the SNc (50–70%) is necessary before the classical motor symptoms of PD become clinically apparent [2,3], suggesting that it may be possible to detect and halt disease progress before clinical symptoms become too severe. Examinations of human brains with PD demonstrate an increase in reactivity of microglia, the resident immune cells of the brain, compared to non-PD age-matched brains [4,5,6], though whether this represents a reactionary process of inflammation or a causative role of microglia in PD pathology is still under investigation.

Photobiomodulation therapy (PBM) involves the application of lasers to biological tissue at wavelengths near the infrared-visible light boundary. In several animal models of PD, including the MPTP and 6-OHDA models, rodents and primates treated with PBM demonstrate attenuated dopaminergic cell loss compared to disease-only animals [7,8,9,10]. The mechanism by which PBM asserts its neuroprotective effect in these models is unclear, though it has been thought to act on cytochrome c oxidase in the mitochondrial electron transport chain [11], and to change the conformational state of key proteins in the nervous and immune systems [12]. More recent studies have highlighted that neuroprotective effects of PBM in normal aging and PD models may be due to a reduction in astrocyte and microglial activation and increased expression of trophic factors such as GDNF [13,14,15] and BDNF [16]. These studies provide support for PBM as a promising candidate for translation to the clinic where regular treatments may slow or prevent further neurodegeneration in patients diagnosed with PD.

Lipopolysaccharide (LPS), a component of the cell membrane of Gram-negative bacteria, is used experimentally as a potent and specific microglial activator [17], increasing cytokine release and changing the functional morphology of the cell [18], which creates a concatenating effect of immune activation. This self-perpetuating cycle of inflammation creates a cellular stress response which can cause the release of reactive oxygen species highly implicated in the pathogenesis of PD, leading to the death of dopaminergic cells which are particularly sensitive to oxidative stress [19,20]. LPS has therefore become of interest in determining the contribution of microglial reactivity to PD-like pathology in animals. Intracranial microinjection of LPS results in 35–70% dopaminergic cell loss in the SNc as early as 16–24 h post-injection in female [21] and male [22] Wistar rats and in male Sprague–Dawley rats [23,24,25], which is associated with a preceding increase in microglia exhibiting a more reactive rod-like or amoeboid morphology [26]. Interestingly, PBM has been shown to reduce LPS-induced microglial activation in vitro by inactivating toll-like receptor 4 [27], suggesting that PBM may be neuroprotective against parkinsonian aetiological factors upstream of mitochondrial distress. However, no studies have yet explored the effectiveness of PBM in an in vivo LPS model of PD, which would provide additional evidence for the effectiveness of PBM in a neuroimmune PD model and provide further clues as to the mechanism of the therapy.

The present experiment aimed to determine whether dopaminergic cell loss in the SNc, following local inflammation and microglial activation by virtue of LPS microinjection in the male Sprague–Dawley rat, would be attenuated by transcranial PBM. Supranigral injections of 10 µg or 20 µg LPS were used, allowing the effectiveness of PBM to be tested at two different dopaminergic lesion sizes. This was chosen since the effectiveness of PBM seems to decrease with increased severity of the PD model [28], though interestingly PBM is more effective in more severe injuries in models of traumatic brain injury [29]. Additionally, we aimed to measure the effect of PBM on motor behaviour using the cylinder, adjusted stepping, and rotarod tests of motor deficit to determine whether any protection of dopamine neurons corresponds to behavioural improvement.

We hypothesised that LPS injection would result in a dose-dependent dopaminergic cell loss in the ipsilateral SNc compared to the contralateral side; that cell loss would be significantly attenuated by PBM in both 10 µg and 20 µg doses, with the potential for complete recovery in the 10 µg, but not 20 µg, dose; that local microglia reactivity would be significantly increased in both doses; and that PBM would improve motor function across three behavioural paradigms implemented. 

## 2. Materials and Methods 

### 2.1. Animals

All experimental procedures have been reported in accordance with the ARRIVE guidelines for research involving animals (https://www.nc3rs.org.uk/arrive-guidelines). Experiments were approved by the University of Sydney Animal Ethics Committee (approval 2016/1101) and adhere to the Australian Code for the Care and Use of Animals for Scientific Purposes (2013) and the Animal Research Act (1985). Male Sprague–Dawley rats (*n* = 41) weighing 210–240 g were sourced from the Animal Resources Centre (Perth, Australia). Rats were group-housed and kept at 22 ± 1 °C with a 12:12 reversed light-dark cycle (lights on at 21:00, lights off at 09:00). Standard chow and water were provided ad libitum. Rats were allowed to acclimate to conditions for four days. 

### 2.2. Experimental Design

**Figure 1:**
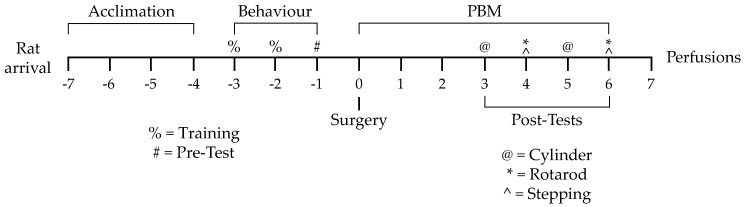
Experimental timeline from rat arrival to perfusion and tissue extraction. Numbers represent days from surgery day. PBM: transcranial near-infrared photobiomodulation.

After four days of acclimation, rats underwent two days of training on three behavioural tasks, followed by a day of baseline pretesting. The next day, rats were randomly allocated into one of three experimental groups: vehicle-only injection with sham PBM (vehicle), LPS-only injection with sham PBM (LPS), or LPS injection with PBM treatment (PBM). Rats underwent intracranial microinjection surgery with 10 µg or 20 µg LPS—or vehicle only—injected supranigrally. Rats were given 7 days of PBM or sham treatment beginning on surgery day. Behavioural post-testing was carried out on post-surgery days 3–6. On post-surgery day 7, rats were perfused and brain tissue was extracted for immunohistochemical analysis. Full details of the methods are elaborated in the following sections. A summary of this experimental timeline is provided in Figure 1.

### 2.3. Behavioural Training and Pretesting

Behavioural training occurred for two days, with pretesting on the third day. Three paradigms designed to measure degree of lateralised motor dysfunction (cylinder test, adjusted stepping test) or overall motor coordination (rotarod) were used. 

The cylinder test is used in PD rat models to quantify motor asymmetry after lateralised SNc lesions and/or treatments [25,30]. The number of rears on the side of the cylinder favouring one leading forepaw or the other gives an indication of lateralised motor deficits. Rats were placed into a Perspex cylinder of diameter 15 cm and height 31 cm. The experimenter left the room and the rat’s behaviour was video-recorded for five minutes.

The adjusted stepping test utilises the tendency of rats to reposition their paw when dragged over a flat surface; fewer repositions have been observed in the paw contralateral to a dopaminergic lesion [25,30,31]. Rats were held with one forepaw restrained, and the unrestrained paw was dragged along a 50 cm length of table at a fixed speed. This was repeated for both forepaws in both forehand and backhand directions. Two replicate trials were conducted in each testing session per rat.

The rat rotarod (Ugo Basile, cat. no. 47700) was used to ascertain general motor coordination. Decreased time spent on the rotarod (latency to fall) is associated with motor impairment [25,32]. Rats underwent two days of training on the device. For pretesting, rats underwent three replicate trials accelerating from 5 rpm to 35 rpm over 5 min, and the mean time elapsed before falling off the device or reaching the end of the 5-minute program was taken for each trial.

### 2.4. Intracranial Microinjection Surgery

The microinjection surgery was performed under isoflurane anaesthesia. The rat was placed on a stereotactic frame and given s.c. lignocaine. An incision was made along the midline of the skull. The skin was reflected and the connective tissue removed to reveal bregma. A Hamilton syringe with a 31-gauge infusion cannula was lowered through a burr hole made with a dental drill to 1 mm above the SNc at the stereotactic coordinates 4.8 mm posterior to bregma, 2 mm left of midline, and 7.6 mm below the dura, based on the atlas of Paxinos and Watson [33] and previously used by Iravani et al. [26]. Next, either 10 µg (*n* = 16) or 20 µg (*n* = 25) of LPS/vehicle was injected. Bone wax was used to fill the burr hole, the skin was sutured closed and a s.c. dose of 0.05 mg/kg buprenorphine was given. 

In the first experiment, vehicle rats (*n* = 8) were injected with 0.9% sterile saline while LPS rats (*n* = 4) were injected with 2 µL of 5 µg/µL (10 µg total dose) LPS (Calbiochem/Merck; serotype O55:B5) in 0.9% sterile saline. PBM animals (*n* = 4) were administered the same dose of LPS as the LPS-only rats. No animals were exposed to PBM without LPS injection since previous studies have shown no effect on dopaminergic cell viability by PBM alone [8,10,28]. The injection was dispensed over 2 min and the needle was left in place for 2 min. In a second experiment, vehicle rats (*n* = 7) were injected with 4 µL 0.9% sterile saline with 1% *w/v* copper(II) phthalocynanine (Monastral blue dye; Sigma, St. Louis, MO, USA) whereas LPS (*n* = 9) and PBM (*n* = 9) rats were injected with 4 µL of 5 µg/µL (20 µg total dose) LPS (Sigma; serotype O26:B6) in 0.9% sterile saline with 0.3% *w/v* dye. The dye was used to visualise the injection site. The decision to vary the dye concentration between groups was made because of the dye’s differential solubility between solutions. The injection was dispensed over 8 min, with the needle left in place for 2 min. 

### 2.5. PBM and Post-Surgery Behavioural Testing

A handheld light-emitting diode (LED) device (Quantum Devices WARP 10) was used for PBM treatments. Device information is available in Table 1. The manufacturer describes the device as outputting 670 nm light with an irradiance of 50 mW/cm^2^ at the aperture. To measure the actual peak wavelength, spectral bandwidth, and surface irradiance, the PBM device was fixed 1 cm from a detector interfacing with a spectroradiometer (OL 756; Optronic Laboratories, Orlando, FL, USA). Irradiance was measured for each integer wavelength from 500 nm to 800 nm and averaged over three trials. The area under the curve (GraphPad Prism, GraphPad Software, Inc.; La Jolla, CA, USA) gave the total irradiance of the device. Available irradiance measurements and treatment parameters are shown in Table 2 following the PBM reporting recommendations of Jenkins and Carroll [34]. A previous study using this device demonstrated that approximately 10% of the irradiance reaching the skin penetrates to the surface of the brain [8].

Rats were habituated to sham PBM restraint before surgery. Rats were restrained using a towel and the PBM device was held 1 cm above the head. The device was turned on for 88 s in PBM rats and left off for this time for LPS-only rats as a sham treatment. PBM or sham PBM was administered two hours following the completion of the injection, and then twice daily at 09:00 and 18:00 for 6 days.

Following surgery, rats were given two days of recovery before behavioural post-testing began. All procedures for the cylinder, adjusted stepping, and rotarod tests were repeated as per the pre-testing parameters. Cylinder testing occurred in the evening of post-surgery days 3 and 5, immediately preceding PBM; rotarod test in the morning of days 4 and 6, immediately following PBM; and adjusted stepping test in the evening of days 4 and 6.

### 2.6. Tissue Extraction and Sectioning

Seven days after microinjection, following brief CO_2_ narcosis, rats were deeply anaesthetised with 120 mg/kg sodium pentobarbital i.p. (Lethabarb; Virbac, Sydney, Australia) and were perfused transcardially with 0.9% saline (pH 7.4), followed by 4% paraformaldehyde (PFA) in sodium tetraborate buffer (pH 9.6). The rats were then decapitated and the brain was rapidly extracted. Brains were post-fixed in 4% PFA for 1 h and transferred to 30% sucrose solution with 0.05% sodium azide in 0.1M phosphate-buffered saline, pH 7.4 (PBS) for ~3 days to ensure cryoprotection. 

A block of midbrain tissue was cut containing all levels of the SNc. Blocks were pinned through the right hemisphere to allow laterality to be determined in free-floating sections and were sectioned into a 1-in-6 series of 30 µm coronal sections using a freezing microtome (Leica CM3050 S).

### 2.7. Immunohistochemistry

One series of sections was stained for tyrosine hydroxylase (TH), an enzyme expressed specifically by catecholaminergic cells and used as a marker of dopaminergic neurons, while the other was stained for IBA1, a microglial marker. Sections were incubated in PBS with either 1:10,000 anti-TH monoclonal primary antibody produced in mouse (ImmunoStar Cat# 22941) or 1:1000 anti-IBA1 monoclonal primary antibody produced in rabbit (Abcam Cat# ab178846) in PBS. They were then incubated with 1:500 donkey anti-mouse secondary antibody (Jackson ImmunoResearch Labs Cat# 715-065-150) for TH sections and 1:500 donkey anti-rabbit (Jackson ImmunoResearch Labs Cat# 711-065-152) for IBA1 sections, and then with 1:1000 ExtrAvidin peroxidase (Sigma-Aldrich, Castle Hill, NSW, Australia). The proteins were then visualised using a 3,3′-diaminobenzidine (DAB) substrate kit (Sigma or Abcam) as per manufacturers’ instructions, allowing the reaction to run for 3.5 min for TH sections and 5.5 min for IBA1 sections.

Sections were then mounted onto gelatinised slides and dehydrated in an ascending series of ethanol and defatted in histolene before coverslipping with DPX mounting medium (Sigma). Before dehydration, slides with IBA1 sections were counterstained for 3 min with neutral red (Sigma), which dyes lysosomes pink-red, as a general cell body counterstain to allow for easy identification and demarcation of the cell-dense SNc.

### 2.8. Stereological Analysis

Stained sections underwent unbiased, blinded cell counting [35] using a bright-field microscope (Olympus BX53, Tokyo, Japan) interfacing with the optical fractionator module of Stereo Investigator software (MBF Bioscience, Williston, VT, USA). Every SNc-containing tissue section was defined in terms of its rostrocaudal position using the rat brain atlas [33]. The counting parameters were 60 µm × 60 µm counting frame and 15 µm optical dissector height with 0% guard. Since TH+ cells were likely to be higher in density than IBA1+ cells, sections were counted with a grid frame of 130 µm × 130 µm for TH sections (21.4% of total cells), and 90 µm × 90 µm for IBA1 sections (44.7% of total cells). Cell estimates were divided by the area of the tracing of the region of interest for each side of the SNc in each section to give a measure of cell density (cells/mm^2^) and placed according to rostrocaudal level, expressed as distance from bregma (mm).

During counting, IBA1+ cells were divided into two subtypes: those of a classical ‘amoeboid’ morphology, defined by a circular shape with no noticeable protruding processes; and microglia of other cell morphologies, including ramified, deramified, and rod-like morphologies. Representative photomicrographs were taken, white-balanced, and adjusted for brightness.

### 2.9. Behaviour Analysis

In the cylinder test, the number of times the rat led a rear with the ipsilateral paw compared to the contralateral paw was counted post hoc using Solomon Coder software (beta version 17.03.22, solomoncoder.com). To normalise for total activity in the cylinder, the number of contralateral paw-lead rears was expressed as a percentage of the sum of all lateralised rears. Each rat’s performance at two time points was expressed as the difference from its baseline (post-surgery time point performance–baseline performance). One rat from the PBM group was excluded from these analyses since it failed to produce any rears on one of the post-surgery days. 

Twenty microgram LPS rats had their rotarod latency to fall normalised to percentage of baseline for group performance comparisons, while raw fall latencies (s) were used for Pearson’s correlation coefficient analyses. Ten microgram LPS rats were not measured at pretest, and therefore were not normalised to baseline.

### 2.10. Statistical Analysis

Stereological cell counts were analysed with one-way ANOVA with Tukey multiple comparisons test after verifying their normality with the D’Agostino-Pearson test (all *p* > 0.05). Pearson’s correlation coefficient was used to correlate anatomical and behavioural measures. Behavioural results were baseline-corrected and underwent repeated-measures two-way ANOVA with experimental group (3: vehicle, LPS, PBM) and time point (3: baseline, post-test 1, post-test 2) factors examined. Post hoc testing was conducted with Tukey’s multiple comparisons test. Analyses were conducted using GraphPad Prism v7.0.2 with significance threshold α = 0.05. Graphs represent mean ± standard error of the mean (SEM). In the 10 µg LPS experiment, the effect size (f) and power (1−β error probability) were calculated using G*Power.

## 3. Results

### 3.1. Ten Microgram LPS Paradigm

**Figure 2:**
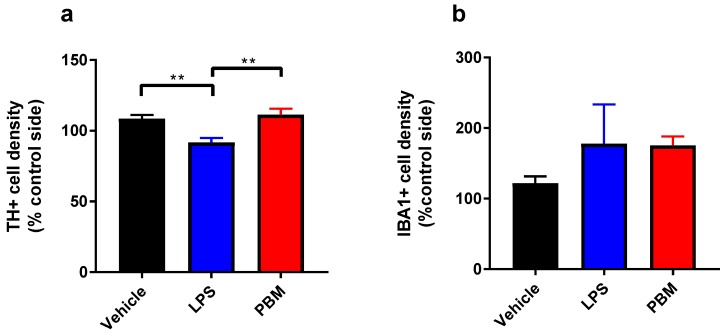
The effects of 10 µg lipopolysaccharide (LPS) and PBM treatment on TH+ and IBA1+ cell densities in the substantia nigra pars compacta (SNc). (**a**) TH+ cell density was significantly reduced from vehicle levels in LPS rats (** *p* < 0.01) and between LPS and PBM rats (** *p* < 0.01). (**b**) There were no significant differences between groups in IBA1+ cell density. Analyses were conducted with one-way ANOVA with Tukey multiple comparisons test. Vehicle: *n* = 8; LPS: *n* = 4; PBM: *n* = 4. Values represent means ± SEM.

Weight loss is a common concomitant of LPS-induced sickness behaviour in rats, however 10 µg LPS led to no decreases in weight in the LPS or PBM groups. There was a significant difference in TH+ cell density in the SNc between groups (*F*_2,*13*_ = 8.928; *p* < 0.01, Figure 2a). TH+ cell density was significantly lower, by ~15%, in the LPS group compared to vehicle (*p* < 0.01), demonstrating that 10 µg LPS was sufficient to cause a small TH+ cell lesion. The TH+ cell density in PBM rats was comparable to vehicle rats and was significantly higher than the LPS-only group (*p* < 0.01). Despite the small number of animals in the LPS and PBM groups (*n* = 4), the effect size (*f*) of PBM treatment was 1.06 and the calculated post hoc power (critical *F*_2,*13*_ = 3.806; 1 - β error probability) was 0.93. This suggests that the experiment was sufficiently powered given the large effect size and low variability (vehicle: *SD* = 7.35; LPS: *SD* = 6.30; PBM: *SD* = 8.34). PBM was therefore successful in protecting these rats from a 15% dopaminergic cell loss. IBA1+ cell density did not differ significantly between vehicle, LPS and PBM groups (Figure 2b). For the cylinder test, there were no significant differences between groups in the proportion of contralateral rears in the cylinder test at post-surgery day 3 or 5. The rotarod test did not uncover any group differences at post-surgery day 4 or 6 in latency to fall off the rotarod. In the adjusted stepping test, there was no significant interaction or treatment group effects for any paw or directionality. These three behavioural tests therefore could not detect the effect of a 15% SNc TH+ cell lesion on motor performance following 10 µg LPS.

### 3.2. Twenty Micrograms LPS Paradigm

Vehicle rats briefly lost a small amount of weight following surgery (<10% of pre-surgery body weight) that recovered after three to four days. There was a significant interaction between time and treatment between groups (*F*_8,*216*_ = 4.975; *p* < 0.0001). Rats in the LPS group lost significant weight compared to vehicle rats on post-surgery days 4–6 (all *p* < 0.05). PBM rats weighed significantly less than vehicle rats from post-surgery day 2 (all *p* < 0.05). There were no differences between LPS and PBM at any time point.

Rats in all groups were hyperphagic and had increased motor activity approximately 3–4 h following surgery, but by 24 h after surgery LPS and PBM rats displayed reduced motor activity in their home cage. A subset of rats in both LPS and PBM groups had postural differences in which they would lean toward the side contralateral to injection. They would additionally place less weight on their contralateral forepaw. After 48 h, LPS and PBM rats became more sensitive to handling and other stressors, which mostly recovered by day 4 post-surgery. These observations combined with weight loss indicate that 20 µg LPS led to temporary signs of sickness behaviour.

**Figure 3:**
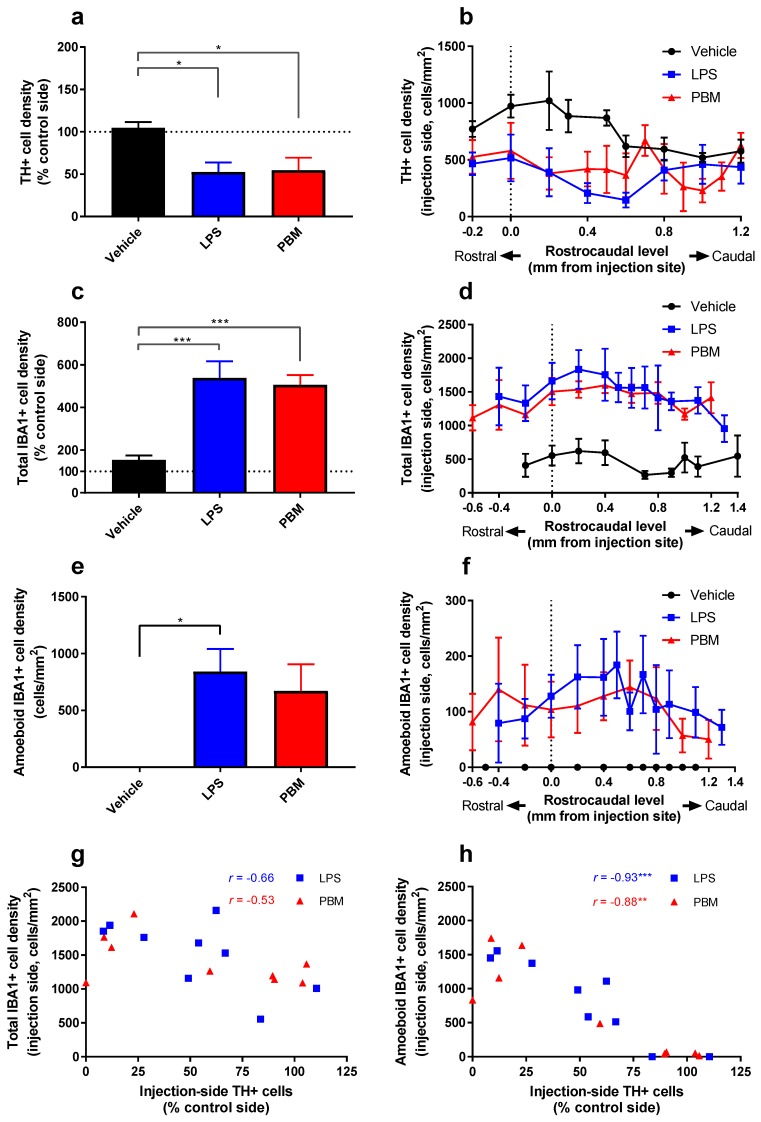
The effects of 20 µg LPS and PBM treatment on TH+ and IBA1+ cell density in the SNc. (**a**) The density (cells/mm^2^) of TH+ cells in the left (injection side) SNc expressed as a percentage of the contralateral (control) side showed a significant difference between LPS and vehicle rats (* *p* < 0.05) and between PBM and vehicle rats (* *p* < 0.05). Both groups had a mean cell loss of ~50%. (**b**) A plot of TH+ cell density in the LPS injection-side SNc, separated by rostrocaudal level and normalised for the level of the injection site in each rat. TH+ cell loss is maximal within ~0.5 mm of the injection site. Each value plotted represents *n* = 3–8 per treatment group. (**c**) IBA1+ cell density ipsilateral to the LPS injection compared to the contralateral side. IBA1+ cell density increases significantly in LPS and PBM rats compared to vehicle rats (*** *p* < 0.001). (**d**) A plot of IBA1+ cell density in the LPS injection-side SNc, separated by rostrocaudal level and normalised for the level of the injection site in each rat. The increase in IBA1+ cells spreads greater than 1 mm from the injection site. Each value plotted represents *n* = 3–9 per treatment group. (**e**) Amoeboid IBA1+ cell density ipsilateral to the LPS injection. There was a significant increase in amoeboid IBA1+ density in the LPS rats compared to vehicle rats (* *p* < 0.05), amounting to almost 50% of total IBA1+ cells, but this did not reach significance for PBM rats. (**f**) A plot of amoeboid IBA1+ cells in the LPS injection-side SNc, separated by rostrocaudal level and normalised for the level of the injection site in each rat. The increase in amoeboid IBA1+ cells is greatest ~0.5 mm caudal to the injection site. Each value plotted represents *n* = 3–9 per treatment group. (**g**) There was no significant relationship between total IBA1+ cell density and TH+ cell density in LPS and PBM rats. (**h**) There was a significant negative relationship between amoeboid IBA1+ cell density and TH+ cell density for both LPS and PBM rats. Analyses for (a,c,e) were conducted with one-way ANOVA with Tukey multiple comparisons test. Vehicle: *n* = 7; LPS: *n* = 9; PBM: *n* = 9. Values represent means ± SEM. Analyses for (g,h) represent correlations with Pearson’s correlation coefficient (** *p* < 0.01, *** *p* < 0.001). LPS: *n* = 9; PBM: *n* = 9.

There was a significant difference in TH+ cell density in the SNc between treatment groups (*F*_2,*22*_ = 5.48; *p* < 0.05). There was a significant decrease between LPS and vehicle rats (*p* < 0.05), and between PBM and vehicle rats (*p* < 0.05), but not between LPS and PBM rats (Figure 3a). This shows that while 20 µg LPS led to a ~50% TH+ cell loss, it could not be protected by PBM. Analysis of expression of TH+ cell density at each rostrocaudal level normalised to the level of the injection site demonstrated that cell loss was most pronounced within 0.5 mm of the injection site, particularly in a caudal direction (Figure 3b). The degree of cell loss, which was more variable (Vehicle: *SD* = 18.16; LPS: *SD* = 33.35; PBM: *SD* = 43.92) than in the 10 µg LPS paradigm (see Section 3.1.), appeared to correspond with the precise location of the injection site in each rat.

There was a significant difference in total IBA1+ cell density between groups (one-way ANOVA; *F*_2,*22*_ = 12.66; *p* < 0.001; Figure 3c). A twenty microgram LPS injection resulted in a 450% increase in IBA1+ microglia compared to vehicle-only injection (*p* < 0.001). PBM rats also displayed a similar increase compared to vehicle rats (*p* < 0.001). There was no significant difference between LPS and PBM rats. Injection site analysis showed that the IBA1+ cell increase is maintained more than 1 mm from the injection site (Figure 3d), suggesting that 20 µg LPS caused a large area of increased microglial reactivity that spreads along the rostrocaudal axis of the SNc.

In measuring microglial cell density, stereological counting distinguished amoeboid-like (reactive) cells and ramified (resting) morphologies. There was a significant difference between groups in the number of amoeboid IBA1+ cells (*F*_2,*22*_ = 4.881; *p* < 0.05; Figure 3e). Compared to vehicle rats, amoeboid IBA1+ cells were significantly more numerous in LPS rats (*p* < 0.05) but not in PBM rats. There was no difference between LPS and PBM rats. Amoeboid IBA1+ cells were rarely found on the contralateral side (LPS: mean = 1.00 cell/mm^2^; PBM: mean = 0.98 cells/mm^2^) and were not present on either side in vehicle rats (mean = 0 cells/mm^2^). Injection site analysis showed that the amoeboid IBA1+ cell increase is greatest 0.5 mm caudal to the injection site (Figure 3f). This pattern strongly correlates with the location of TH+ cell loss (Figure 3b), suggesting a relationship between amoeboid microglia and dopaminergic neuron degeneration.

The correlation between IBA1+ cell density and TH+ cell density was analysed for both LPS and PBM cohorts separately, both for total IBA1+ cell density (Figure 3g) and amoeboid IBA1+ cells (Figure 3h). While there was no significant relationship between total IBA1+ cell density and TH+ cell density in LPS or PBM rats, a significant negative relationship was found in both groups when amoeboid IBA1+ cells were considered (LPS: *r* = −0.93; R^2^ = 0.86; *p* < 0.001; PBM: *r* = -0.88; R^2^ = 0.78; *p* < 0.01). There was no significant relationship between IBA1+ and TH+ cell densities in vehicle rats. Dopaminergic degeneration was therefore strongly associated with the presence of amoeboid microglia rather than total microglia.

**Figure 4:**
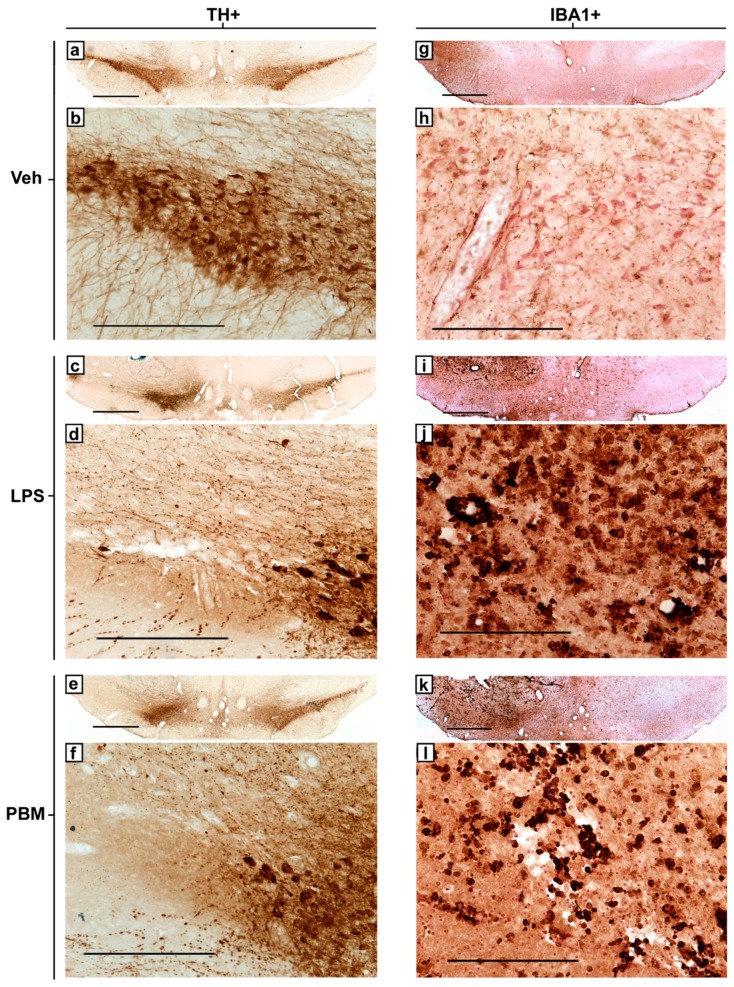
Representative photomicrographs of TH+ and IBA1+ immunostaining in the SNc of 20 µg LPS-treated rats from each experimental group at the same bregma level. (**a**) Bilateral and (**b**) injection-side TH+ staining for a vehicle rat. (**c**) Bilateral and (**d**) injection-side TH+ staining for a representative LPS rat. Large areas of the SNc are without intact cells, and nearby remaining cells increased in their expression of TH. Remaining processes are punctate and dystrophic. (**e**) Bilateral and (**f**) injection-side TH+ staining for a representative PBM rat. (**g**) Bilateral and (**h**) injection-side IBA1+ staining for a vehicle rat. Cells were ramified or rod-like in morphology with long, distinguished processes. Brown staining: immunostaining precipitate. Pink-red staining: neutral red cell body counterstain. (**i**) Bilateral and (**j**) injection-side IBA1+ staining for an LPS-only rat. IBA1+ microglia greatly increased in density and retracted their processes to take on an amoeboid morphology. Regions with complete TH+ cell loss corresponded with regions exhibiting IBA1+ microglia with majority amoeboid morphology. (**k**) Bilateral and (**l**) injection-side IBA1+ staining for a PBM rat, showing similarities to LPS rats with a similar lesion size. TH+ and IBA1+ stained sections for each experimental group were from adjacent sections in the same rat. Bilateral 4× images (a,c,e,g,i,k; scale bar 1 mm) present the SNc of both sides with the injection side on the left, while magnified 20× unilateral (injection-side only) images (b,d,f,h,j,l; scale bar 250 μm) allow for visualisation of cellular morphology.

Representative photomicrographs of TH+ and IBA1+ immunostaining in the injection-side SNc of vehicle, LPS, and PBM rats are presented in Figure 4. The images demonstrate the extent of TH+ cell loss in the LPS-injected ipsilateral side compared to the contralateral side and vehicle-injection (Figure 4a,c,e), leaving behind only dystrophic processes in the most lesioned areas (Figure 4d,f), and the increase in IBA1+ microglial density and amoeboid morphology following 20 µg LPS injection compared to vehicle-injection (Figure 4h,j,l).

**Figure 5:**
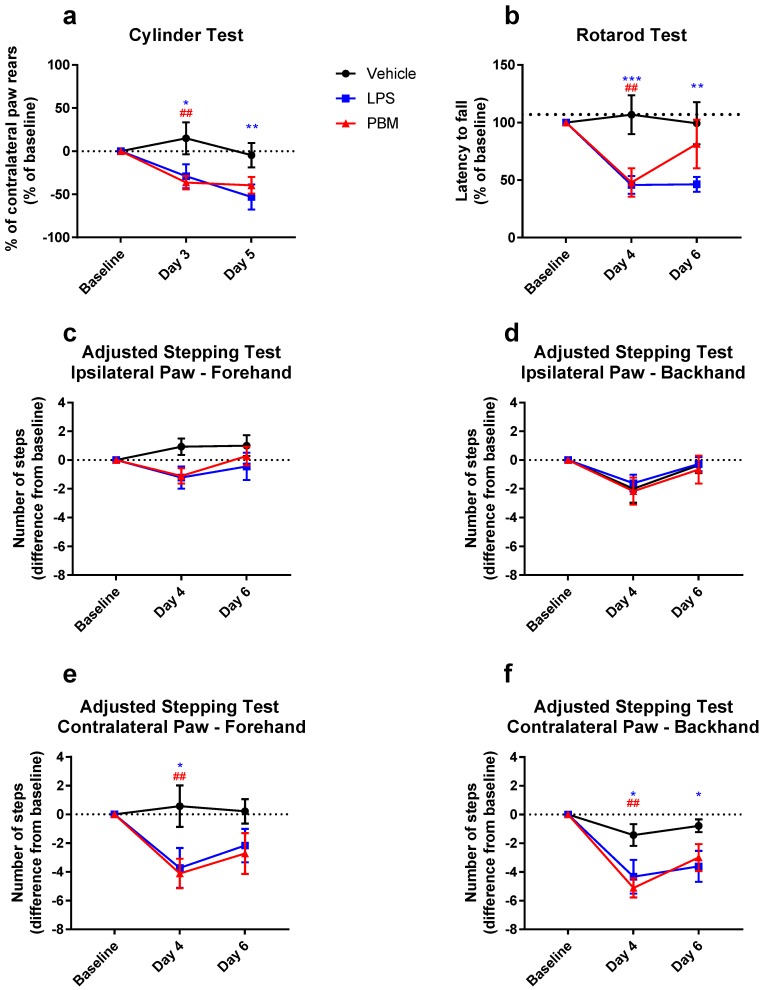
Motor behaviour in the cylinder, rotarod, and adjusted stepping tests before and after 20 µg LPS injection and PBM treatment. (**a**) Proportion of rears led with the paw contralateral to injection in the cylinder test. There was a significant difference compared to vehicle for both LPS (* *p* < 0.05) and PBM (^##^ *p* < 0.01) groups at post-surgery day 3, which persisted for the LPS group at post-surgery day 5 (** *p* < 0.01). Vehicle: *n* = 7; LPS: *n* = 9; PBM: *n* = 8. (**b**) Time taken to fall off the rotarod. There was a significant difference between LPS and vehicle groups at both time points (*** *p* < 0.001 at day 4; ** *p* < 0.01 at day 6) and between PBM and vehicle at post-surgery day 4 only (^##^ *p* < 0.01). PBM rats improved on post-surgery day 6 but were not significantly different from LPS rats. Vehicle: *n* = 7; LPS: *n* = 9; PBM: *n* = 9. (**c**) There was no significant difference between groups in the number of adjusted steps made by rats with the ipsilateral (left) paw in the forehand direction in the adjusted stepping test, nor for the (**d**) ipsilateral paw backhand direction. (**e**) In the contralateral (right) paw, forehand direction, there was a significant difference at post-surgery day 4 between LPS and vehicle groups (* *p* < 0.05) and between PBM and vehicle groups (*^##^ p* < 0.01), but not between LPS and PBM groups. There were no significant differences at post-surgery day 6. (**f**) In the contralateral paw backhand direction, there were significant differences in the number of steps between LPS and vehicle groups at post-surgery days 4 and 6 (* *p* < 0.05) and between PBM and vehicle groups at post-surgery day 4 only (^##^ *p* < 0.01). (c–f): Vehicle: *n* = 7; LPS: *n* = 9; PBM: *n* = 9. (a–f): Repeated-measures two-way ANOVA with Tukey multiple comparisons test. Values represent baseline-corrected means ± SEM.

The mean change in contralateral paw use in the cylinder test is shown in Figure 5a. There was a significant interaction between the experimental group and time point (*F*_4,*42*_ = 2.82; *p* < 0.05) in cylinder test performance. There were significant differences between the vehicle group and both LPS and PBM rats (*p* < 0.05 and *p* < 0.01, respectively) at post-surgery day 3, but not between LPS and PBM groups. At post-surgery day 5, this difference with the vehicle group was maintained with the LPS group only (*p* < 0.01), with no difference between LPS and PBM groups. Twenty microgram LPS therefore decreases contralateral paw rearing behaviour in the cylinder test that PBM is not able to significantly attenuate.

For rotarod performance, there was a significant interaction between the experimental group and time point (*F*_4,*44*_ = 4.44; *p* < 0.01; Figure 5b). By post-surgery day 4, both LPS and PBM rats decreased in their rotarod performance by ~50% compared to vehicle rats (*p* < 0.01 and *p* < 0.001 respectively). There was no difference between LPS and PBM rats at this time point. However, on post-surgery day 6, while LPS rats still performed significantly worse than vehicle rats (*p* < 0.01), PBM rats showed some improvement and were no longer significantly different to vehicle rats. The difference between LPS and PBM rats at day 6 did not reach statistical significance. These results demonstrate that rotarod performance is affected by a 50% TH+ cell lesion size following 20 µg LPS, and PBM may be capable of alleviating this by post-surgery day 6.

In the adjusted stepping test, there was no difference between groups for the ipsilateral (left) paw in the forehand (Figure 5c) and backhand directions (Figure 5d). There was no significant interaction between the experimental group and time point for the contralateral (right) paw in the forehand direction (*F_4,44_* = 2.38; *p* = 0.066), nor was there a significant simple main effect of the experimental group (*F_2,22_* = 2.85; *p* = 0.079), but there was a significant simple main effect of time (*F_2,44_* = 6.79; *p* < 0.01; Figure 5e). Multiple comparisons testing showed significant differences at post-surgery day 4 between vehicle and LPS rats (*p* < 0.05) and between vehicle and PBM rats (*p* < 0.01), but not between LPS and PBM rats (*p* > 0.05). There were no significant differences at post-surgery day 6. There was a significant interaction between the experimental group and time point for the contralateral paw backhand direction (*F*_4,*44*_ = 2.946; *p* < 0.05; Figure 5f), where there were differences at post-surgery day 4 between vehicle and LPS rats (*p* < 0.05) and between vehicle and PBM rats (*p* < 0.01). At day 6 post-surgery, the LPS rats continued to have significantly fewer adjusted steps compared to vehicle (*p* < 0.05), whereas PBM rats were no longer significantly different to vehicle. There were no differences between LPS and PBM groups at either time point. The adjusted stepping test was therefore able to detect a motor deficit in the contralateral paw in the forehand and backhand directions following 20 µg LPS. In this test, PBM treatment appeared to have no significant benefit compared to the LPS only group.

**Figure 6:**
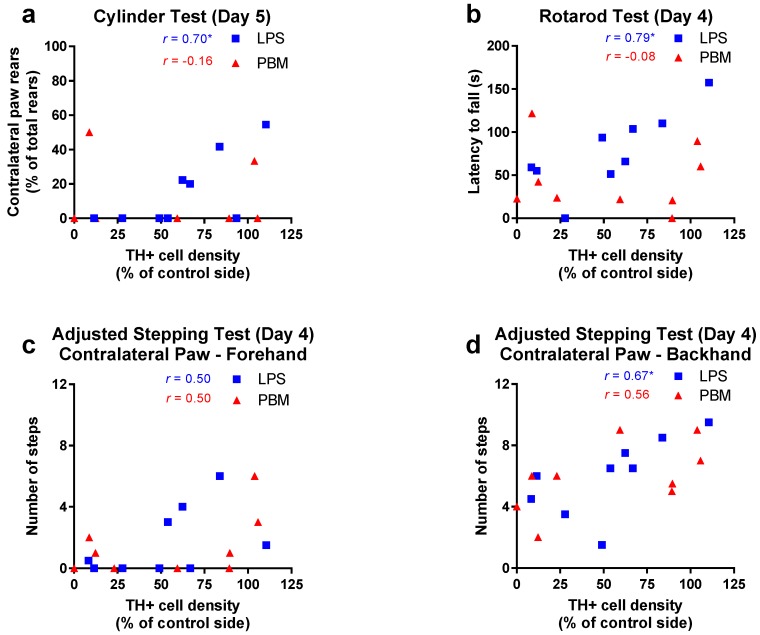
The relationship between TH+ cell density and motor behaviour in the cylinder, rotarod, and adjusted stepping tests following 20 µg LPS injection and PBM treatment. (**a**) A significant positive relationship between TH+ cell density and cylinder test performance was found for the LPS group at post-surgery day 5 (* *p* < 0.05) but not for the PBM group. Vehicle: *n* = 7; LPS: *n* = 9; PBM: *n* = 8. (**b**) A significant positive relationship existed between TH+ cell density and rotarod performance for LPS rats at post-surgery day 4 (* *p* < 0.05), but not the PBM group. Vehicle: *n* = 7; LPS: *n* = 9; PBM: *n* = 9. (**c**) No significant relationship existed between the contralateral (right) paw forehand stepping performance and TH+ cell density for LPS or PBM rats at post-surgery day 4. Vehicle: *n* = 7; LPS: *n* = 9; PBM: *n* = 9. (**d**) A significant positive relationship existed between TH+ cell density and contralateral paw backhand stepping performance for LPS rats at post-surgery day 4 (* *p* < 0.05), but not PBM-treated rats. Vehicle: *n* = 7; LPS: *n* = 9; PBM: *n* = 9. *r* values represent Pearson’s correlation coefficient.

Individual performance on the behavioural tests was compared to the density of TH+ cells in the SNc in both 20 µg LPS and PBM groups. For the cylinder test, at post-surgery day 3, no relationship existed for either LPS or PBM rats, however at post-surgery day 5, there was a significant positive relationship for LPS rats (*r* = 0.70; R^2^ = 0.49; *p* < 0.05) but not PBM rats (Figure 6a). This suggests that once the acute toxic effects of LPS had subsided, PBM treatment dissociated the relationship between SNc TH+ cell density and cylinder test performance which was present in LPS-only rats. In the rotarod test, at post-surgery day 4 there was a significant positive relationship for LPS rats (*r* = 0.79; R^2^ = 0.63; *p* < 0.05) but not for PBM rats (Figure 6b). In the adjusted stepping test, there was no relationship for LPS rats for the contralateral (right) paw in the forehand direction (Figure 6c) but there was a significant positive relationship for LPS rats at post-surgery day 4 in the contralateral paw, backhand direction (*r* = 0.67; R^2^ = 0.45; *p* < 0.05, Figure 6d). Contralateral paw stepping was not significant for PBM rats in either direction.

## 4. Discussion

These experiments aimed to ascertain the ability of near-infrared PBM to attenuate dopaminergic cell loss, as well as concomitant microglial reactivity and motor disturbances, in the LPS model of Parkinson’s disease. It was demonstrated that supranigral LPS was able to induce a dose-dependent loss of dopaminergic cells in the SNc, resulting in 15% cell loss following 10 µg LPS and 50% cell loss following 20 µg LPS. While 10 µg LPS resulted in no detectable motor deficits or increases in microglial density, 20 µg LPS was associated with severe motor deficits and an increase in microglia density. In particular, the number of amoeboid microglia in the SNc was highly correlated with the degree of cell loss. 

PBM was able to fully protect SNc dopaminergic cells from LPS-induced degeneration at 10 µg; however, contrary to our expectations, PBM had no significant neuroprotective effect on dopaminergic neurons or microglial density following 20 µg LPS. Despite no overall protection of dopaminergic cells following 20 µg LPS, PBM did have some positive effects on motor performance that appear independent of the degree of SNc dopaminergic cell loss, suggesting possible extranigral effects of PBM treatment.

A summary of the TH+ and IBA1+ immunohistochemistry results is presented in Table 3. Results pertaining to the behavioural tests are summarised in Table 4.

### 4.1. PBM Neuroprotection

These results provide the first evidence that PBM may be neuroprotective against low-level neurodegeneration in an inflammatory model of PD, protecting against a dose of LPS sufficient to cause 15% dopaminergic cell death, but not a dose resulting in 50% cell death. These findings suggest that there is a threshold of nigral inflammation beyond which PBM ceases to have a beneficial effect on dopaminergic cell survival.

In support of our findings, lesions consisting of 15% cell loss due to MPTP and 6-OHDA injections have been shown to be fully recoverable with PBM [10,36]. Doses causing up to 50% cell loss were not recoverable in mice [28], however, a significant improvement was possible from this lesion size in primates [7]. We found no significant improvement in PBM rats compared to LPS-only rats at larger lesion sizes, however the distribution of TH+ neurons is more bimodal in the PBM group compared to LPS-only rats, suggesting possible neuroprotective effects in some of the PBM rats with smaller lesions. Indeed, it has been well documented that for neuroprotective agents to work they must be administered at a time when the loss of dopaminergic neurons is not too devastating, and consequently neural imaging techniques and biomarkers are being developed and validated to aid early diagnosis clinically [37,38].

Since the TH+ cell loss likely occurred in the first 16–24 h, as has previously been demonstrated [22,26], the timing of the PBM may be critical in its effectiveness in this model. The 12 h between the first and second PBM treatments may be sufficient time to cause irreversible damage in the 20 μg dose. Therefore, consistent neuroprotection by PBM using the current treatment regime relies on a low level of LPS-induced inflammation that results in a relatively small loss of dopaminergic neurons.

### 4.2. Microglial Reactivity

Ten microgram LPS showed no difference in IBA1+ cell density between LPS and PBM rats, suggesting that PBM did not exert its neuroprotective effect by modulating the number of reactive microglia. The primary mechanism of PBM is thought to be by changing the conformational state of cytochrome c oxidase, involved in the mitochondrial electron transport chain [11], though several other mechanisms have been suggested [12]. A dampening effect of PBM on glial activation has been shown in normal aging and in toxin-based models of PD. In particular, the number of reactive astrocytes and microglia was reduced in the striatum of aged mice that had received 8 months of PBM [15], whilst PBM in MPTP-treated primates led to a reduction in astrogliosis in the SNc and striatum and an increase striatal GDNF [13,14]. In an in vitro study, the activation of cultured microglia by LPS was attenuated by PBM at the level of the toll-like receptor that is activated by LPS, suggesting a mechanism for PBM directly involving microglia [27]. Additionally, since neuronal death occurs within 24 h in this model [26], it may be that by post-surgery day 7 microglial activation has subsided, and so is not detectable in the 10 µg experiment reported here. More sophisticated morphological analysis of microglia may shed more light on whether microglia are involved in the PBM protection seen after 10 μg LPS. It may be the case that PBM-related neuroprotection is less effective for microglia with an amoeboid rather than ramified morphology.

A novel finding in these experiments was that the presence of amoeboid microglia within the SNc was necessary for large TH+ lesion sizes. A threshold appears to exist for dopaminergic cell death such that a small proportion of neurons are susceptible to the oxidative stress caused by the release of pro-inflammatory mediators from IBA1+ cells with a reactive, but not amoeboid, morphology [39], as was the case following 10 μg LPS. After this point, further cell death to the more stress-resistant population requires the phagocytic, amoeboid microglia phenotype seen after 20 μg LPS. An inflammatory environment sufficient to induce microglia to take on this amoeboid morphology is also most conducive to dopaminergic cell loss. These findings confirm functional phenotypes of microglia both as potential targets for treatment as well as markers for pathological inflammation in PD.

### 4.3. PBM and Motor Behaviour

None of the behavioural tests used in this study had the sensitivity required to demonstrate motor deficits after a 15% SNc lesion following 10 µg LPS, although this is not surprising given that this represents a subclinical proportion of cell loss in humans [2,3]. However, all tests reliably demonstrated reduced performance following 20 μg LPS that led to a 50% lesion. Our findings are also in keeping with Sharma and Nehru [24], who observed that a 60% LPS lesion in Sprague–Dawley rats resulted in a 50% reduction in latency to fall off the rotarod.

A trend in the rotarod test was the improvement of PBM rats at the second post-surgery time point (day 6) compared to LPS rats. The rotarod test measures not only motor coordination, but also factors such as motivation to engage with the task, which relates to LPS-induced inflammation [40]. Several rats were observed to simply allow themselves to fall off the device on some or all of their trials; this is perhaps an unsurprising finding since motivation is a frequent confound in rotarod testing [41]. The rotarod was the only one of our three motor tests that showed an appreciable improvement in the PBM group compared to the LPS-only group, and it may be explained, at least in part, by an effect of PBM on motivation or other factors important in this task.

Though it would be expected that motor performance in individual rats would negatively correlate with dopaminergic lesion size in both LPS-only and PBM groups, correlation analysis demonstrated that the relationship between dopaminergic cell density and motor performance broke down for PBM-treated rats (see Figure 6). That is, dopaminergic cell density explains the variability in performance in the cylinder, stepping and rotarod tests in LPS rats, but not PBM rats. It has been previously reported that PBM can result in behavioural improvements in the absence of corresponding dopaminergic cell survival [42], but also can protect cells while having little effect on motor behaviour [10]. PBM may therefore have diverse effects on behaviour in animals with dopaminergic lesions and interfere with the otherwise strong relationship between nigral cell density and motor performance in this model. Since PBM increases mitochondrial ATP, and the PBM dose likely reached most of the brain, it is interesting to speculate whether other brain circuitries were affected energetically by PBM differently between rats, leading to this dissociation via an extranigral pathway. The exact mechanism behind this change warrants further investigation but may involve neuroplasticity in the motor cortex.

### 4.4. Considerations of the LPS Model and Study Limitations 

The animal model chosen to investigate the efficacy of PBM in attenuating parkinsonian dopaminergic depletion depends critically on relating a proposed aetiology of the affliction to the putative mechanism of action of PBM (see the review by Duty and Jenner [43] for a summary of animal models of PD, their mechanisms, and their utility, including the LPS model). While the LPS model has proven useful in replicating the immunological hallmarks of PD against which PBM may have a therapeutic mechanism beyond its direct mitochondrial effect [12,44], the results of the present investigation suggest key considerations for its implementation in future studies.

LPS doses of 10 µg and 20 µg required to achieve 15% and 50% lesion sizes, respectively, are inconsistent with previous reports. Iravani and colleagues [26] demonstrated that a 10 µg LPS injection of the same volume, serotype, manufacturer, and supranigral injection coordinates as the present study, albeit in Wistar rats, achieved a 70% lesion size compared to the 15% achieved here. Sharma and Nehru [24], in a 5 µg intranigral LPS injection in Sprague–Dawley rats, achieved a 60% SNc TH+ lesion size at day 21 post-injection. Hsieh, et al. [45] required a 30 µg intranigral LPS dose to significantly damage the nigrostriatal pathway in Sprague–Dawley rats. The inconsistency in lesion size between laboratories reveals the need for investigators to establish a dose–response curve for their LPS microinjection procedures in a particular strain of rodent in order to achieve a lesion size appropriate for investigating the efficacy of PD treatment options. 

The degree of lesion size variation following 20 µg LPS was larger than expected, however some of this was accounted for by plotting the location of injection sites relative to the SNc. Other potential explanations for the variability in lesion size are individual differences between rats in the susceptibility of dopamine cells to LPS-induced cell death, or the volume of LPS reaching the SNc, which may have been inconsistent due to dorsal spreading along the injection tract. Injection of the volume directly into the SNc, rather than supranigrally, may reduce some of this variation in lesion size, however this risks direct mechanical damage to neurons.

The experiments reported here used only male rats, although the LPS model has been established in females [21]. Moreover, PBM is neuroprotective in an α-synuclein overexpression model of PD in females [46]. Despite this, sex differences exist in microglial phenotype and reactivity properties [47,48] and motor behaviour on tests such as the rotarod [49]. Therefore, the neuroprotective effects of PBM in the LPS model reported here may not generalise to females and should be investigated by future studies.

While the density and morphology of microglia in relation to the observed dopaminergic cell loss were investigated in these experiments, additional mechanistic pathways—such as the involvement of cytokines, astrocytes, and trophic factors such as BDNF and GDNF—were not. Future studies may investigate these to develop a clearer understanding of the mechanism of action of PBM in the LPS model.

### 4.5. Clinical Prospects of PBM 

These results provide additional support that PBM may be a useful therapy in slowing or halting the progression of PD in humans in the future. It would, however, rely on early diagnosis using biomarkers or neural imaging that are sensitive enough to detect preclinical PD so that PBM can be used to halt progression before clinical symptoms appear. The relative thickness of the human skull, and the depth from the skull surface at which the SNc resides in humans, means that transcranial application of PBM, as used in the present study, is unlikely to exert a therapeutic effect directly in the midbrain [50]. However, light may reach overlying cortical brain regions and exert extranigral effects. PBM devices have been developed in monkeys that can be surgically implanted either directly into brain tissue or into the lateral ventricle to allow for better penetration and effective dose [7,14]. There is evidence that PBM applied peripherally (e.g., to the abdomen) can protect against dopaminergic cell damage in a mouse model, though not as well as direct transcranial application [28]. Exciting ‘light bucket’ trials in Tasmania, Australia, involving a wearable transcranial PBM device, have shown promise [51], and formal clinical trials are currently underway. 

## 5. Conclusions

These experiments provide the first evidence of the neuroprotective effects of PBM in a neuroinflammatory rat model of PD, thus contributing further to PBM as an exciting prospect for neuroprotection against early to mid-stage PD in humans. Although PBM caused no significant motor improvement at the group level with a large lesion, motor performance at the individual level no longer correlated to the lesion size following PBM treatment, suggesting extranigral motor improvements in some animals. More research is required regarding optimal dosimetry of the therapy and the mechanisms by which it asserts its neuroprotective and motor effects if it is to make a convincing transition to the clinic.

## Figures and Tables

**Table 1:** A summary of the PBM device information.

Device Information	
Manufacturer	Quantum WARP Light Devices
Model	Quantum Light WARP 10
Number of emitters	48
Emitter type	LED
Spatial distribution of emitters	48 emitters spaced in a square grid pattern 5 mm apart within a circular aperture
Beam delivery	Handheld

**Table 2:** A summary of the PBM irradiation and treatment parameters. All measurements were conducted by the researchers unless otherwise noted.

**Irradiation Parameters**	
Centre wavelength [nm]	675
Spectral bandwidth [nm]	23
Operating mode	Continuous wave
Average Radiant Power [mW]	500 ^^^
Irradiance at aperture [mW/cm^2^]	50 ^^^
Beam shape	Circular
Beam profile	Gaussian
**Treatment Parameters**	
Irradiance at target [mW/cm^2^]	40.84
Exposure duration [s]	88
Radiant exposure [J/cm^2^]	3.594
Radiant energy [J]	35.94
Number of points irradiated	1
Area irradiated [cm^2^]	10
Application technique	Held 1 cm above skin
Number of treatments	13
Total radiant energy [J]	467.2

^^^ Per manufacturer’s measurements.

**Table 3:** A summary of the main immunohistochemical cell counting results from the 10 µg and 20 µg LPS experiments. Arrows represent a significant difference compared to vehicle rats in the specified direction; hyphens represent no significant difference from vehicle rats.

Cell Count Measure	10 µg LPS		20 µg LPS	
	LPS	PBM	LPS	PBM
TH+ cell density (% contralateral side)	↓	-	↓	↓
IBA1+ cell density (% contralateral side)	-	-	↑	↑

**Table 4:** A summary of the main behavioural testing results from the 10 µg and 20 µg LPS experiments. Arrows represent a significant difference compared to the performance of that group at baseline; hyphens represent no significant difference compared to baseline performance.

Behaviour Measure		10 µg LPS						20 µg LPS					
		Post-Test 1			Post-Test 2			Post-Test 1			Post-Test 2		
		Veh	LPS	PBM	Veh	LPS	PBM	Veh	LPS	PBM	Veh	LPS	PBM
Cylinder		-	-	-	-	-	-	-	↓	↓	-	↓ ^^^	-
Rotarod		-	-	-	-	-	-	-	↓ ^^^	↓	-	↓	-
Stepping	IF ^1^	-	-	-	-	-	-	-	-	-	-	-	-
	IB ^2^	-	-	-	-	-	-	-	-	-	-	-	-
	CF ^3^	-	-	-	-	-	-	-	↓	↓	-	-	-
	CB ^4^	-	-	-	-	-	-	-	↓ ^^^	↓	-	↓	-

^^^ Behavioural measure significantly correlated with TH+ cell density as measured at post-surgery day 7. ^1^ IF: ipsilateral paw, forehand direction. ^2^ IB: ipsilateral paw, backhand direction. ^3^ CF: contralateral paw, forehand direction. ^4^ CB: contralateral paw, backhand direction.
